# Screening and monitoring of travellers returning from countries affected by Bundibugyo virus: an overview of European approaches, July 2026 

**DOI:** 10.2807/1560-7917.ES.2026.31.28.2600578

**Published:** 2026-07-16

**Authors:** Jake Dunning, Erica Binetti, Arne B Brantsæter, David Ekqvist, Sabiena G Feenstra, Jocelyn J Herstein, David K Kayembe, F-Xavier Lescure, Agata Mikolajewska, Annelies Mondelaers, Marta Mora-Rillo, Chantal Rovers, Laura Scorzolini, Corien M Swaan, Francesco Vairo, Erika Vlieghe, Timo Wolf, Luca Zweers, Emanuele Nicastri, on behalf of the European HLIU Network, and of the ESCMID Emerging Infection Subcommittee

**Affiliations:** 1 Pandemic Sciences Institute, University of Oxford, Oxford, United Kingdom; 2 Centre for Respiratory Infection, Imperial College, London, United Kingdom; 3 National Institute for Infectious Disease Lazzaro Spallanzani IRCCS, Rome, Italy; 4 Department of Infectious Diseases and Department of Acute Medicine, Oslo University Hospital, Oslo, Norway; 5 Clinical Department of Infectious Diseases in Östergötland, Region Östergötland, Linköping, Sweden; 6 RIVM Centre for Infectious Disease Control, Bilthoven, the Netherlands; 7 University of Nebraska Medical Center, College of Public Health, Omaha, Nebraska, United States; 8 National Public Health Institute (INSP), Ministry of Public Health, Kinshasa, Democratic Republic of the Congo; 9 Emerging Infections Subcommittee, European Society of Clinical Microbiology and Infectious Diseases (ESCMID), Basel, Switzerland; 10 Infectious and Tropical Diseases Department, APHP, Bichat Hospital and Université Paris Cité, INSERM, IAME, Paris, France; 11 Robert Koch Institute, Centre for Biological Threats and Special Pathogens (ZBS), Strategy and Incident Response (ZBS 7), Clinical Management and Infection Control (ZBS 7.1), Berlin, Germany; 12 Department of General Internal Medicine, Infectious Diseases and Tropical Medicine, Antwerp University Hospital, Antwerp, Belgium; 13 Global Health Institute, University of Antwerp, Antwerp, Belgium; 14 High Level Isolation Unit, La Paz University Hospital, IdiPAZ, CIBERINFEC, Madrid, Spain; 15 Goethe University Frankfurt, University Hospital, Department of Internal Medicine II, Infectious Diseases, Frankfurt am Main, Germany; 16 Department of Internal Medicine, Radboud University Medical Center, Nijmegen, the Netherlands; 17 European High-Level Isolation Units (HLIU) Network, Europe; 18 Emerging Infections Subcommittee, European Society of Clinical Microbiology and Infectious Diseases (ESCMID), Basel, Switzerland

**Keywords:** Bundibugyo virus, Ebola virus, European Union, public health policies, public health preparedness, risk assessment

## Abstract

The 2026 Ebola outbreak caused by Bundibugyo virus in the Democratic Republic of the Congo and Uganda has prompted European countries and the United States to revise measures for travellers, healthcare workers and humanitarian personnel returning from affected areas. We compare current procedures and protocols with those implemented during the 2013–2016 Ebola outbreak. Despite some national differences, policies have largely converged towards risk-based management, early case detection, rapid isolation, exposure-based monitoring and healthcare preparedness, rather than routine border screening.

The 2026 outbreak of Ebola disease caused by Bundibugyo virus (BDBV) in the Democratic Republic of the Congo (DRC) and Uganda represents a major public health concern. The outbreak is unfolding in a fragile setting marked by large population movements with intense cross-border movement to neighbouring countries, humanitarian instability and ongoing armed conflict, all of which have complicated surveillance and containment efforts. Officially declared on 15 May 2026, after several weeks of undetected transmission, the 17th Ebola disease outbreak in DRC originated in Ituri Province (north-eastern DRC), North and South Kivu (eastern DRC) [1-3]. Its continued progression is of particular concern given the limited knowledge of this orthoebolavirus species and the scarcity of available medical countermeasures. The initial diagnostic focus on Ebola virus (*Orthoebolavirus zairense*) delayed identification of BDBV (*Orthoebolavirus bundibugyoense*) and the implementation of appropriate control measures, contributing to healthcare-associated transmission and infections among healthcare workers [4].

Here, we outline the measures in place in Europe and the United States (US) as in June 2026 and compare them with those adopted during the 2013 to 2016 Ebola virus outbreak, using publicly available information together with information shared by public health and infectious disease experts from different countries collaborating on preparedness for high-consequence infectious diseases within the newly formed European High-Level Isolation Unit network.

## Risk of international spread and preparedness for case importation

As with other orthoebolaviruses, transmission occurs primarily through direct contact with infected body fluids or contaminated materials: the risk increases during advanced stages of illness and unsafe burial practices, while individuals are not considered infectious before symptom onset. In the absence of specific therapies and vaccines for BDBV infection, the cornerstones of outbreak response are case detection and management, laboratory confirmation, isolation, infection prevention and control measures, contact tracing and community engagement. However, surveillance remains challenging in affected areas [1-4].

Although the likelihood of Bundibugyo virus disease cases being diagnosed outside the two outbreak-affected countries in Africa is low [5], the risk is not zero [6], as demonstrated by a recent imported case in France involving a humanitarian healthcare worker returning from the DRC [7], and all countries must have plans for the management of imported cases. Importation may occur through different pathways, and preparedness should address several scenarios, including (i) the planned medical evacuation or repatriation of individuals with suspected or confirmed infection or specific high-risk exposures to BDBV, (ii) the arrival and prompt management of infected travellers returning directly from affected areas, and (iii) indirect importation through unrecognised secondary transmission chains involving intermediary countries, which may delay case recognition. These distinct scenarios require different operational responses. Accordingly, many European countries and the US have developed entry procedures and contact management protocols for travellers, healthcare workers, and humanitarian personnel returning from Ebola-affected regions. 

## National approaches

In the European Union/European Economic Area and the United Kingdom (UK), national policies show a high degree of convergence (Table). The countries covered here rely on early case recognition, exposure-based monitoring, prompt isolation of suspected cases, and management at designated referral centres, rather than routine entry screening for people returning from BDBV-affected countries. Returning travellers are advised to self-monitor for symptoms for 21 days and seek medical care promptly if symptoms develop. Healthcare workers and humanitarian personnel are monitored using a risk-based approach. Exposure level is determined according to potential exposure and use of personal protective equipment. Quarantine is only recommended for individuals with high-risk exposures [5,8]. Coordination between public health authorities, occupational health services, humanitarian organisations and referral hospitals is a common feature of preparedness strategies throughout European countries covered here.

**Table:** Border control measures and monitoring protocols for travellers, healthcare workers and humanitarian personnel during the 2013–2016 Ebola virus outbreak and the 2026 Bundibugyo virus outbreak, Europe and United States

**Country/area**	**2013–2016 approach**	**Registration before departure from outbreak-affected countries (2026)**	**Reporting upon return from outbreak-affected countries (2026)**	**Quarantine (2026)**	**Symptom monitoring (2026)**	**Ability of low-risk contacts to work (2026)**
EU / ECDC [8]	Promoted risk-based, proportionate measures and avoided blanket quarantine	No EU-wide requirement	No EU-wide requirement	Only for high-risk exposures	Active monitoring for high risk; self-monitoring for low risk	Allowed if asymptomatic, unless high-risk exposure
Belgium [9]	Passenger locator form and airport screening; followed ECDC guidance; coordination between NGOs, academia, public health authorities and reference centres	Yes (for HCWs through NGOs/employers)	Reporting according to occupational health and local public health procedures	Only for high-risk exposures	Risk-based according to exposure assessment	Allowed if asymptomatic, unless high-risk exposure
France [10]	Airport questionnaires and screening at points of entry; collaboration with NGOs	No	Not specified	Only for high-risk exposures	Risk-based according to exposure assessment	Allowed if asymptomatic, unless high-risk exposure
Germany [11]	Enhanced entry procedures and questionnaires at major airports	No	Yes (self-declaration to public health authorities strongly recommended)	Only for high-risk exposures	Risk-based according to exposure assessment	Allowed if asymptomatic, unless high-risk exposure
Italy [12]	Variable airport screening and questionnaires at points of entry; followed ECDC guidance	Yes (for HCWs, pre-departure notification by NGOs)	Yes (mandatory self-declaration to public health authorities)	Only for high-risk exposures	Risk-based according to exposure assessment	Allowed if asymptomatic, unless high-risk exposure
The Netherlands [13]	No routine entry screening; followed ECDC guidance	Yes (for HCWs through NGOs/academic institutions)	Reporting according to occupational health and local public health procedures	Only for high-risk exposures	Risk-based according to exposure assessment	Allowed if asymptomatic, unless high-risk exposure
Norway [14]	No routine entry screening; emphasis on preparedness and surveillance	No	No routine reporting	Not routinely required; isolation if symptoms develop	21-day self-monitoring for individuals with relevant exposure	Not specifically addressed in public guidance
Spain [15,16]	Enhanced controls after the secondary transmission in hospital setting in Madrid; conservative monitoring of exposed HCWs	No	Not specified	Only for high-risk exposures	Risk-based according to exposure assessment	Allowed if asymptomatic, unless high-risk exposure
Sweden [17]	No routine entry screening; emphasis on preparedness and surveillance	No	Reporting according to occupational health and local public health procedures	Only for high-risk exposures	Risk-based according to exposure assessment	Allowed if asymptomatic, unless high-risk exposure
United Kingdom [18]	Enhanced screening at major airports and Eurostar (international rail service); risk-stratified follow-up	Yes (Returning Workers Scheme)	Yes	Only for high-risk exposures	Risk-based according to exposure assessment	Allowed if asymptomatic, unless high-risk exposure
United States [19]	Enhanced screening and routing through designated airports	No	Temporary US entry restriction for all travellers in DRC and for certain travellers in Uganda or South Sudan in the previous 21 days. Limited case-by-case exceptions	For travellers authorised to enter the US, movement restrictions depend on the individual exposure-risk assessment	21-day risk-based symptom monitoring with public health follow-up	According to exposure-risk category

CDC: US Centers for Disease Control and Prevention; HCW: healthcare worker; NGO: non-governmental organisation; ECDC: European Centre for Disease Prevention and Control; EU: European Union; US: United States.

Sources: Information on current preparedness measures (2026) was obtained from publicly available documents issued by national public health authorities, Ministries of Health, ECDC and CDC [8-13,15-19]. Information on the 2013–2016 Ebola virus outbreak was obtained from publicly available guidance and official documents available during that outbreak and supplemented by information provided by national public health and infectious diseases experts participating in the European High-Level Isolation Unit Network. No formal requests for information were made to national health authorities specifically for this table.

## Assessment and management of returning healthcare workers in the 2026 outbreak

Despite this overall convergence, some operational differences remain in how countries assess and manage returning healthcare workers. Across all countries, returning healthcare workers undergo occupational risk assessment, with the possibility of temporary work restrictions based on exposure level; however, the specific criteria and follow-up requirements vary by country. 

Belgium has a formal guideline with no formal notification requirements between non-governmental organisations (NGOs) and regional health authorities, but relies on coordination between occupational health services and local public health authorities for the assessment and follow-up of returning healthcare workers [9].

France relies heavily on coordination between humanitarian organisations, healthcare institutions, and public health authorities; individuals returning from the epidemic areas remain under self-monitoring and are advised to refer to seek medical advice if symptoms develop [10]. 

Germany strongly recommends self-declaration to public health authorities for all healthcare workers who were involved in the care of Ebola disease patients in outbreak-affected areas. For those with unprotected or inadequately protected high-risk exposures, early consultation of the deploying organisation or the public health authority with the Permanent Working Group of Competence and Treatment Centres for high consequence infectious diseases (STAKOB; www.rki.de/stakob) centre is recommended for risk assessment and consideration of appropriate measures [11]. 

Italy places particular emphasis on maintaining clinical vigilance through mandatory self-declaration to regional health authorities and may monitor high-risk individuals in high-level isolation facilities [12]. 

The Netherlands has well-established coordination among NGOs, academic institutions, and healthcare organisations to facilitate the follow-up of returning healthcare workers and students. Returning healthcare workers are managed through occupational health and local public health procedures. [13]. 

Norway does not require routine reporting upon return, and quarantine is not routinely recommended. Individuals who develop symptoms are advised to isolate and contact healthcare services. The ability to work is not specifically addressed in the public guidance [14].

Spain adopts a risk-based approach, with 21-day self-monitoring for travellers returning from affected areas. Returning healthcare workers undergo exposure-based risk assessment and follow-up, with active monitoring and temporary work restrictions for significant exposures [15,16].

Sweden relies primarily on public health surveillance systems and exposure-based follow-up, with returning healthcare workers managed according to occupational health and local public health procedures [17].

The UK operates a Returning Workers Scheme, originally established for the 2013 to 2016 Ebola virus outbreak, which provides monitoring and support for returning humanitarian and healthcare workers [18].

In contrast, the US has adopted a more restrictive strategy. Temporary entry limitations have been introduced for all travellers recently in the DRC, with case-by-case humanitarian and law enforcement exceptions, and for certain travellers who have been in South Sudan or Uganda during the previous 21 days. For travellers authorised to enter the US, access is restricted to designated airports and includes mandatory health screening procedures upon arrival, with in-person evaluation by personnel from the Centers for Disease Control and Prevention. Asymptomatic travellers may continue to their destination under symptom monitoring for 21 days, whereas symptomatic individuals may be transferred to a hospital for isolation and diagnostic evaluation [19].

## Discussion

Compared with the 2013–2016 Ebola virus outbreak, when several European countries implemented heterogeneous airport entry screening measures, current practice has largely converged towards a harmonised risk-based approach, symptom monitoring, exposure-based follow-up and healthcare preparedness rather than routine border screening. Taken together, European policies in the countries covered here broadly align with European Centre for Disease Prevention and Control's (ECDC) core principles, although some differences persist in operational procedures and institutional roles. Unlike the US, the European approach prioritises rapid clinical recognition and isolation, and exposure-based monitoring over indiscriminate quarantine. Consistent with World Health Organisation (WHO) and ECDC guidance, European countries presented here have favoured targeted surveillance and clinical readiness over general travel restrictions, routine arrival screening, or quarantine of asymptomatic travellers without high-risk exposure. These important aspects of the European response, support proportionate public health action while limiting unnecessary disruption.

At the same time, recently announced exit measures by the DRC Government [20], which include administrative requirements and mandatory waiting periods for individuals leaving outbreak-affected areas, may have implications for the deployment of humanitarian staff and healthcare workers. This issue warrants careful consideration, given the need to balance measures aimed at limiting the risk of disease spread with the effectiveness of the humanitarian outbreak response.

Beyond the implementation of measures in outbreak-affected countries, effective coordination and preparedness are also needed for the management of individuals returning to their country of residence from areas with ongoing transmission. Further alignment of European approaches, building on existing ECDC guidance, could help promote common standards in specific aspects such as pre-travel counselling, point-of-entry information, 21-day symptom surveillance, criteria for active vs passive follow-up, proportionate work restrictions based on exposure risk, and referral pathways to designated centres. Such alignment may be particularly useful for the management of international healthcare workers and individuals returning after visiting family members in affected countries, whose exposures may be close, heterogeneous and not always adequately documented.

## Conclusion

As outlined above, European countries surveyed here broadly share a common approach based on individual risk assessment. Further alignment on specific aspects, such as reporting requirements, referral pathways, follow-up criteria, and work restrictions, could facilitate the cross-border management of humanitarian workers, while respecting national autonomy and the specificities of each country’s legal framework and healthcare system. Such alignment could enhance preparedness and proportionate risk assessment while ensuring freedom of movement, healthcare workforce sustainability, and the timely detection and management of suspected imported cases. In parallel, sustained investment in raising awareness and strengthening knowledge among healthcare professionals is essential to ensure the consistent implementation of these strategies and maintain long-term preparedness. Together, these measures would additionally strengthen Europe's capacity to respond effectively to future outbreaks of emerging high-consequence infectious diseases.

## Data Availability

No new datasets were generated or analysed in this study. All information used in this manuscript is derived from publicly available sources referenced in the article.
